# EXPLORING THE HEALTH CARE UTILIZATIONS AND OUTCOMES OF FAMILY CAREGIVERS FOR OLDER ADULTS WITH DEMENTIA

**DOI:** 10.1093/geroni/igae098.3130

**Published:** 2024-12-31

**Authors:** Yu-Qi Cheng, Ying-Chun Li

**Affiliations:** National Sun Yat-sen University, Kaohsiung, Kaohsiung, Taiwan (Republic of China); National Sun Yat-sen University, Kaohsiung, Kaohsiung, Taiwan (Republic of China)

## Abstract

Family caregivers of older adults with dementia usually face more negative issues, such as family relationships, personal career, financial status, and mental and physical problems. Therefore, family caregivers could increase healthcare utilizations and worsen healthcare outcomes. Nevertheless, previous studies results were inconsistent, which could be due to different health policies or healthcare systems in the studied countries. Whether family caregivers receive better care and reduce their burdens under Taiwan’s universal healthcare system with free access to healthcare. This is a very important policy research question. We linked outpatient data, inpatient records, medical care facility factors, and registration files from the National Health Insurance Program Data to form the analyzed datasets. Family caregivers for people over 65 with dementia were identified in 2008 and followed for five years. As a result, Family caregivers of older persons with dementia faced long time pressure which led to an increased probability of diseases, such as depression, anxiety, and chronic diseases (all were p< 0.01). These caregivers with disease disorders also significantly consumed more total, outpatient, inpatient, and emergency expenditures (all were p< 0.01). In addition, caregivers for those with dementia who had low and middle-income status used more medical resources (OR=2.16, p< 0.01) and had worse mental health outcome (OR=1.11, p< 0.01). In summary, the empirical results indicated that family caregivers who stay in high stress for a longer time are more likely to cause physical and mental health problems. These family caregivers will use more healthcare resources and experience poorer healthcare outcomes.

